# Influence of nanoparticle-mediated transfection on proliferation of primary immune cells *in vitro* and *in vivo*

**DOI:** 10.1371/journal.pone.0176517

**Published:** 2017-05-02

**Authors:** Susanne Przybylski, Michaela Gasch, Anne Marschner, Marcus Ebert, Alexander Ewe, Gisa Helmig, Nadja Hilger, Stephan Fricke, Susanne Rudzok, Achim Aigner, Jana Burkhardt

**Affiliations:** 1 Fraunhofer Institute for Cell Therapy and Immunology (IZI), Leipzig, Germany; 2 Translationszentrum für Regenerative Medizin (TRM), University of Leipzig, Leipzig, Germany; 3 Institute for Clinical Immunology, University of Leipzig, Leipzig, Germany; 4 Rudolf-Boehm-Institute for Pharmacology and Toxicology, Clinical Pharmacology, University of Leipzig, Leipzig, Germany; Universidad de Castilla-La Mancha, SPAIN

## Abstract

**Introduction:**

One of the main obstacles in the widespread application of gene therapeutic approaches is the necessity for efficient and safe transfection methods. For the introduction of small oligonucleotide gene therapeutics into a target cell, nanoparticle-based methods have been shown to be highly effective and safe. While immune cells are a most interesting target for gene therapy, transfection might influence basic immune functions such as cytokine expression and proliferation, and thus positively or negatively affect therapeutic intervention. Therefore, we investigated the effects of nanoparticle-mediated transfection such as polyethylenimine (PEI) or magnetic beads on immune cell proliferation.

**Methods:**

Human adherent and non-adherent PBMCs were transfected by various methods (e.g. PEI, Lipofectamine^®^ 2000, magnetofection) and stimulated. Proliferation was measured by lymphocyte transformation test (LTT). Cell cycle stages as well as expression of proliferation relevant genes were analyzed. Additionally, the impact of nanoparticles was investigated *in vivo* in a murine model of the severe systemic immune disease GvHD (graft versus host disease).

**Results:**

The proliferation of primary immune cells was influenced by nanoparticle-mediated transfection. In particular in the case of magnetic beads, proliferation inhibition coincided with short-term cell cycle arrest and reduced expression of genes relevant for immune cell proliferation. Notably, proliferation inhibition translated into beneficial effects in a murine GvHD model with animals treated with PEI-nanoparticles showing increased survival (p_PEI_ = 0.002) most likely due to reduced inflammation.

**Conclusion:**

This study shows for the first time that nanoparticles utilized for gene therapeutic transfection are able to alter proliferation of immune cells and that this effect depends on the type of nanoparticle. For magnetic beads, this was accompanied by temporary cell cycle arrest. Notably, in GvHD this nonspecific anti-proliferative effect might contribute to reduced inflammation and increased survival.

## Introduction

Gene therapy is a promising therapeutic option in modern medicine. Target cells are transfected with nucleic acids to enhance, suppress or correct the expression of a specific gene [1]. In the last 20 years, various transfection methods have been established and optimized for clinical application in the treatment of viral infections such as HIV [2] or cancer [3,4]. Compared to viral delivery systems, non-viral vectors provide distinct advantages such as reduced risk of insertional mutagenesis and potentially low toxicity, ease of chemical synthesis and preparation also at larger scale, as well as high delivery capacities (reviewed in [5]). Non-viral transfection techniques can be divided into physical and chemical gene delivery methods. One physical transfection method is magnetofection. A strong magnetic field is applied to introduce iron oxide particles loaded with nucleic acids into target cells [6]. Thereby, transfection can be achieved by magnetic sedimentation and increased endolysosomal uptake [7]. Chemical transfection methods are based on lipids (liposomes) or cationic polymers that form condensed complexes with the negatively charged nucleic acids through electrostatic interactions (reviewed in [5]). Nucleic acids are protected from degradation, and cellular uptake and intracellular gene delivery is improved by complexing reagents. To further increase of transfection efficacy, nanoparticular transfection methods can also be combined [8,9]. Despite the enormous progress in research and development of non-viral cell transfection methods, the delivery of nucleic acids into primary cells, particularly into immune cells, is still challenging. On the other hand, immune cells are highly relevant targets for gene therapeutic approaches, e.g. in the promising field of immune oncology, however it has been difficult to reach primary immune cells using non-viral gene transfer delivery systems because of low transfection rates, cell toxicity or possible induction of apoptosis [10,11].

Efficient transfection of human primary T lymphocytes *in vitro* has been performed mostly by electroporation to introduce DNA [12], RNA [13] or small interfering RNA into the target cell [14]. However, electroporation is not suitable for systemic *in vivo* application. Nanoparticle-based methods, such as cationic polymers, are particularly promising for transfection of primary immune cells. For example, DEAE-dextran was successfully applied for transient transfection of primary murine B lymphoblasts [15]. Besides cationic polymers, lipid-based transfection reagents (e.g. Lipofectamine^®^, FuGene) have been used to introduce genetic information into human dendritic cells (DC) in order to modify and enhance DC-mediated T lymphocyte activation [16]. It is also possible to couple transfection particles to tissue or cell specific antibodies, therefore allowing targeted gene therapy. Previously, this was done in primary human T cells, using anti-CD3-coupled polyethylenimine (PEI) for receptor-mediated endocytosis [17].

Several studies showed that transfection efficiency of cationic lipids [18,19], cationic polyplexes like PEI or polypeptides [20] is influenced by the cell cycle phase and the mitotic activity of the transfected cell. It is also reasonable to assume that the effect could be reciprocal and transfection might non-specifically influence cellular functions such as proliferation. In case of immune cells, initiation of proliferation upon a stimulus is one of the most important cornerstones of cellular immunity. Thus, transfection methods influencing proliferation might also impact basic immune function, especially *in vivo*.

Investigations of immune system effects often rely on PBMCs peripheral mononuclear blood cells (PBMCs) *in vitro*, which are an easily accessible cell population representing the high complexity of the immune system more closely than established tumor cell lines. In fact, PBMCs consist of different immune cell subpopulations, including monocytes as well as T and B lymphocytes. These populations show substantial differences in the uptake rates of gene therapeutics, e.g. depending on phagocytotic activity, and impaired proliferative capacity might influence basic immunological function of cells [21]. Moreover, the utilization of primary cells avoids artifacts associated with transformation and long-term culture of cell lines.

While the impairment of cellular functions upon transfection will usually be associated with negative outcomes, it may also be beneficial. A devastating systemic immunological disorder is graft versus host disease (GvHD), a severe complication of hematopoietic stem cell transplantation that is caused by exuberant immune response of transplanted donor T cells against host antigens, resulting in severe systemic inflammation and possibly death. This prompted us to study possible nanoparticle effects in the context of GvHD. To this end, we made use of a GvHD animal model that allowed an *ex vivo* approach in an otherwise systemic disease, since the stem cell transplant that contains overreacting T cells may be treated prior to transplantation [22].

Thus, we investigated the influence of nanoparticle-based transfection reagents on the proliferation of primary human PBMCs and non-adherent PBMCs (i.e., without monocytes) and observed distinct differences. To further explore a possible *in vivo* relevance, polycationic PEI transfection particles were applied to a murine model of the systemic immune reaction GvHD to assess whether transfection non-specifically influences disease progression *in vivo*.

## Material and methods

### Human PBMC isolation, cell culture and separation of non-adherent PBMC

Buffy coats were obtained from anonymous healthy human donors with written consent obtained by staff at the Institute of Transfusion Medicine, University Leipzig, where consent forms are stored. Information on date of blood sampling, gender, infection status and blood type for each donor is also stored anonymized at laboratory facilities of Dr. Burkhardt. The local ethics committee approved this procedure and the use of the obtained buffy coat (file number 272-12-13082012). PBMCs were isolated by density gradient centrifugation with Ficoll-Hypaque (density: 1.077 g/ml; Pan Biotech, Aidenbach, Germany). PBMCs were cultured in a humidified incubator in RPMI 1640 (Gibco, Darmstadt, Germany) supplemented with 10% FCS at 37°C and 5% CO_2_. Non-adherent PBMCs (naPBMCs, containing mostly lymphocytes and NK cells but not monocytes) were separated from total PBMCs by incubating PBMCs in a culture flask for 2 hours at 37°C and 5% CO_2_ and were harvested by gentle pipetting. Viability was monitored by counting Trypan blue (Sigma-Aldrich, Taufkirchen, Germany) stained cells.

### Transfection and stimulation

PBMCs or naPBMCs were seeded at 2.5 x 10^5^ cells per well in a 96 well cell culture plate and transfected with branched low molecular weight polyethylenimine (4–10 kDa PEI-F25 LMW [23]; hereafter referred to as “PEI”) at 2.5 μg/well, Ibafect at 3 μg/well (PromoKine, Heidelberg, Germany), or Lipofectamine^®^ 2000 at 1.5 μg/well (Life Technologies, Darmstadt, Germany), each diluted in X-Vivo media (Lonza, Cologne, Germany). Transfection chemicals were complexed with a nonsense antisense oligonucleotide (AON, 2’O-Me-PTO-RNA, 5’-caaggcgauuacacuaccu-3’, 0.5 μg/ 2.5 x 10^5^ cells) labeled with Fluorescein amidite (FAM), or a nonsense siRNA (5’-cguacgcggaauacuucga-3’, 0.5 μg/ 2.5 x 10^5^ cells) labeled with Alexa488 (both Eurofins Genomics, Ebersberg, Germany) to measure uptake rates by flow cytometry 24h after transfection. Magnetofection was done with 1 μl Matra-A suspension per well (PromoKine, Heidelberg, Germany). For combined chemical-magnetofection, transfection reagents were pre-incubated with iron oxide nanoparticles. PEI was premixed with FluidMag at a ratio of 1:1000 (1 μg/well), Ibafect premixed with MA Lipofection Enhancer (0.5 μg/well, PromoKine) and Lipofectamine with CombiMag (0.5 μg/well, Chemicell, Berlin, Germany) for 20 minutes at room temperature. For magnetofection, a cell monolayer was formed by mixing cells with Matra-S Immobilizer (100:3 v/v; Promokine) for 15 min prior to seeding and submitting thus treated cells to a magnetic field (universal magnet plate, PromoKine) for 15 minutes at room temperature. This was repeated when introducing the magnetofection-oligonucleotide mix. After 24h, cells were washed twice and stimulated with phytohaemagglutinin (PHA, 5 mg/ml, Sigma Aldrich, Munich, Germany) for 24-48h prior to lymphocyte proliferation transformation test (LTT).

### Lymphocyte proliferation transformation test (LTT)

^3^H-thymidine (1 μCi/well, 20 μCi/ml, Amersham Bioscience, Hartmann Analytik GmbH, Braunschweig) was added to cells 24h post transfection. ^3^H-thymidine genomic integration correlating to released β radiation was measured after 16-18h with a Wallac 1450 MicroBeta^®^ TriLux (PerkinElmer, Waltham, MA, USA) and is documented as CCPM (cell counts per minute).

### Cell cycle analysis

Cell cycle progression was analyzed by applying the APC BrdU Flow kit (BD Biosciences, Heidelberg, Germany) according the manufacturer´s protocol. Influence on cell cycle stages was monitored for transfection of Matra-A beads alone in the T cell line RLD1 (ACC-415, obtained from the DSMZ), and for PEI or Ibafect with and without magnetic beads in human PBMCs. Briefly, BrdU solution (10 μM) was added to 5 x 10^5^ transfected and stimulated cells for up to 6 days. On days 0, 1, 3 and 6, cells were stained with anti-BrdU-APC and 7-AAD and analyzed by flow cytometry. BrdU^-^ / 7-AAD^-^ cells refer to G0/G1 phase, BrdU^+^ cells refer to S-phase, and BrdU^-^/7-AAD^+^ cells refer to G2/M phase of the cell cycle.

### Analysis of mRNA expression of proliferation genes

Total RNA was isolated with peqGOLD TriFast (Peqlab, Erlangen, Germany) and transcribed into cDNA using the RevertAid H Minus First Strand cDNA Kit (ThermoScientific, Waltham, MA, USA) according to the manufacturer´s protocol. Primers for the proliferation marker gene *Ki67* and the lymphocyte proliferation specific *Il2* cytokine as well as the housekeeping gene *Rplp0* were used (Table 1). Real-time PCR was performed in quadruplicates (N_PBMC donors_ = 4) on a QuantStudio^™^ 6 Flex Real-Time PCR System (Life Technologies, Darmstadt) with 95°C for 15min, followed by 50 cycles of 95°C for 15s, 60°C for 20s, and 72°C for 30s. The relative mRNA expression was calculated compared to *Rplp0* expression by using the ΔΔCt-method. Samples transfected with or without magnetic particles were also compared with significance calculated by Student’s T test.

**Table 1:** PCR primer sequences.

target gene	sequence
*Rplp0*	for: GGCGACCTGGAAGTCCAACT rev: CCATCAGCACCACAGCCTTC
*Mki67 (also Ki67)*	for: CCAGCTTCCTGTTGTGTCAA rev: AGCCGTACAGGCTCATCAAT
*Il2*	for: CCCTTGCTAATCACTCCTCA rev: GAGCTCCTGTAGGTCCATCA

### GvHD mouse model

All animal experiments were approved by the Regional Board of Animal Care (animal experiment registration number TVV 53/12, Saxony, Germany) and mice were housed, treated and handled in accordance with the guidelines of the University Leipzig Animal Care Committee. Donor C57Bl/6^wt^ mice and recipient Balb/c^wt^ mice were purchased from Charles River (Sulzfeld, Germany). For GvHD induction, healthy male Balb/c^wt^ mice (8–12 weeks old) were irradiated with 8 Gy per mouse (X-Ray apparatus D3225, Orthovoltage) [24]. Bone marrow cells and splenocytes were isolated from healthy male C57Bl/6^wt^ mice which were euthanized humanely by CO_2_ fumigation. Splenocytes (2 x 10^7^ cells) were incubated with PEI (50 μg/ 2 x 10^7^ cells; PEI-Co; N = 16) and nonsense AON (10 μg) or with Ibafect (30 μg/ 2 x 10^7^ cells; Iba-Co; N = 3) and MA Enhancer (10 μg/ 2 x 10^7^ cells) and nonsense AON (10 μg) for 4h at 37°C and 5% CO_2_. Untreated cells were used as control (GvHD-Co; N = 16). Splenocytes were washed and added to bone marrow (BM) cells in a 1:1 ratio. Transplantation of splenocyte-BM cells into irradiated Balb/c recipient mice was performed intravenously into the tail vein in 150 μl 0.9% NaCl. Irradiation control was injected with cell-free NaCl (NaCl-Co; N = 4). To reduce suffering, animals were short-term anesthetized by ether during the transplantation procedure.

Mice were weighted and scored daily as described previously [25] (also see Table 2).

**Table 2:** GvHD animal score.

criteria	score 0	score 0.5–1	score 1.5–2
weight loss	< 10%	> 10% up to < 25%	> 25%
posture	healthy	humpbacked when sitting	strongly humpbacked, impaired movements
mobility	healthy	slightly impaired mobility	motionless unless stimulated
fur	healthy	slightly untended	strongly untended
skin	healthy	scabby at tail and paws	visible naked skin

Briefly, 5 criteria (weight, mobility, posture, skin and fur conditions) were evaluated daily with healthy animals scoring 0 points and affections being rated up to 2 points per item up to a cumulated maximum of 10 points. Mice were humanely euthanized by CO_2_ fumigation when reaching a cumulative score of >6 or when extensive impositions became apparent such as food refusal or a weight loss of more than 25%. One animal in the GvHD-control group died unexpectedly (i.e. without reaching a critical score) on day 12 and one in the PEI group on day 25. All four NaCl-treated not transplanted control animals died at day 12. It is known from literature and accepted by the Regional Board of Animal Care as part of the approved experimental protocol that within this model, animals may die suddenly, usually within the first two weeks, due to direct consequences of irradiation such as hematopoietic insufficiency.

Besides clinical scoring, about 100 μl blood was drawn from the retro-bulbar venous plexus every two weeks by glass capillary. To reduce stress for the animals, they were short-term anesthetized by ether as allowed by exception permit issued to the manager of the Leipzig University animal care facility where animals were housed and approved as part of the experimental protocol by the Regional Board of Animal Care. To simulate treatment of transplanted patients, mice were also given antibiotics continuously (25 mg/kg Baytril [Enrofloxacin] at 1.5 ml 10% solution in 1000 ml drinking water).

Lymphocyte subpopulations (CD4^+^, CD8^+^, CD3^+^, CD19^+^, CD25^+^) in blood were analyzed by flow cytometry according to [24]. Survival was documented by Kaplan Meier curve (SPSS, version 22) and significance calculated by Log-Rank (Mantel-Cox)-Test.

## Results

### PBMCs and naPBMCs proliferation is affected by nanoparticle-based transfection reagents

**Fig 1:**
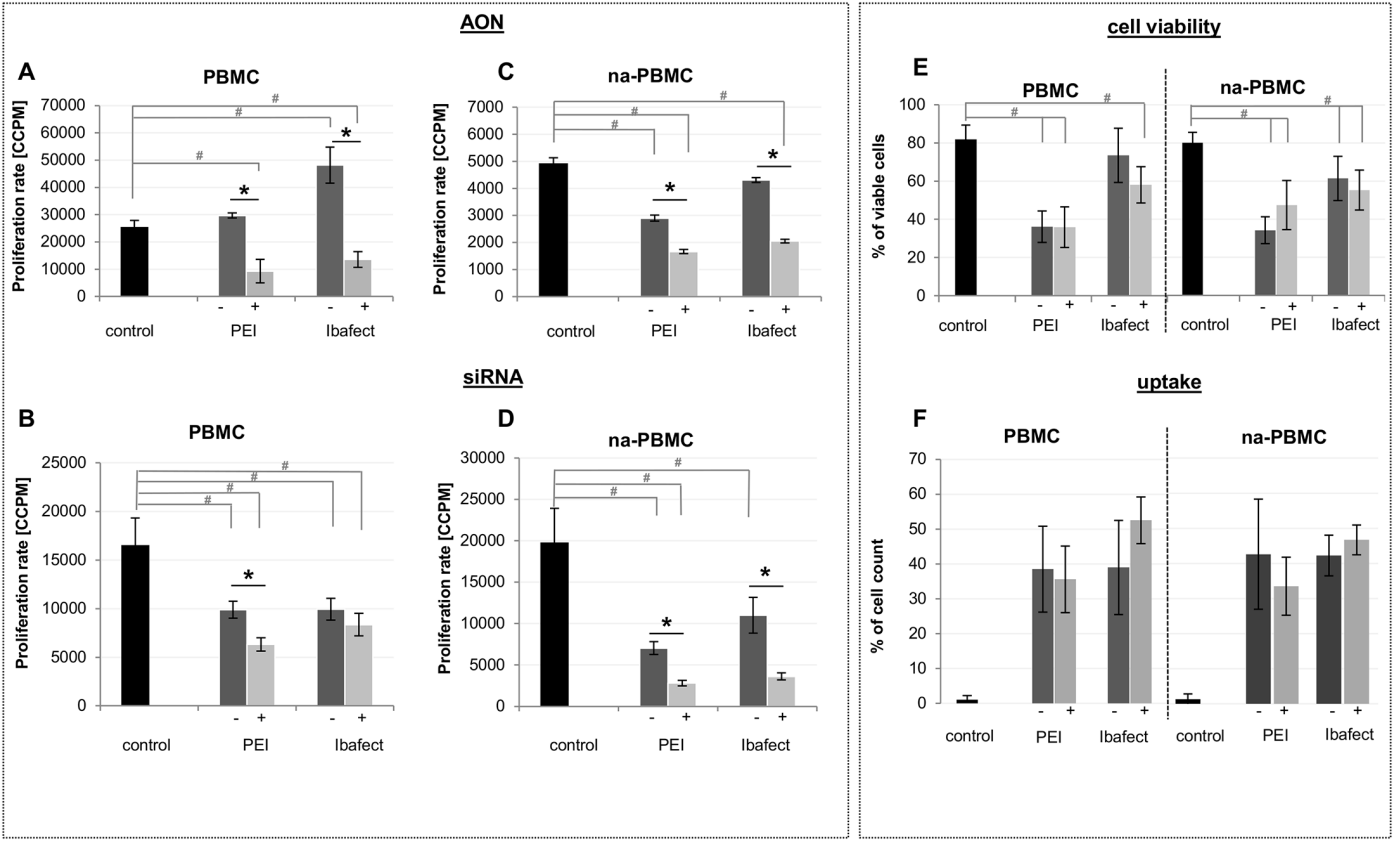
Effect of transfection on proliferation of primary human immune cells w/o iron oxide nanoparticles (-); with particles (+). PBMCs or non-adherent PBMCs (naPBMCs) were transfected with FAM-labeled nonsense AON (2’O-methyl-PTO-RNA) (A, C) or Alexa488 labeled nonsense siRNA (B, D) with or without magnetic iron oxide nanoparticle transfection enhancement (dark gray = without, light gray = with particles) by applying PEI (+/- FluidMag) and Ibafect (+/- MA Enhancer). Controls were untransfected cells (black bar). Cells were washed and stimulated with PHA (0.05 mg/ml) 24h post transfection. Proliferation was measured by LTT. Briefly, incorporation of ^3^H thymidine (20 μCi/ml) was measured 16-18h post stimulation as cell count per minute (CCPM) and data is shown as mean ± SE (N_PBMC donor_ = 4). Also shown are cell viabilities measured by Trypan blue staining (E) and uptake rates measured by flow cytometry (F) 24h after transfection as mean ± SD. Significance levels were calculated by Student´s T test, * p < 0.05 between transfected groups, # p < 0.05 compared to respective control.

Proliferation was significantly increased in PBMCs transfected with nonsense AON and liposomal Ibafect (p = 0.0013, N = 4, Fig 1A) or Lipofectamine (p = 4x10^-5^, N = 4, data not shown). Both chemicals showed nearly doubling proliferation rates (increase by 89.1% and 96.4%, respectively). In contrast, the use of the polycationic polymer PEI for AON transfection without iron oxide particle enhancement did not significantly alter PBMC proliferation (Fig 1A). Interestingly, this was dependent on the nucleic acid since reduced proliferation was measured upon PEI mediated transfection of nonsense siRNA into PBMCs (Fig 1B). This proliferation inhibitory effect was also found in the case of AON or siRNA transfected into naPBMCs (Fig 1C and 1D).

The addition of magnetic particles to potentially enhance transfection efficacy was associated with a significant decrease of proliferation compared to untransfected control cells in all instances. This was true for all tested methods comparing standard transfection and iron oxide particle enhanced transfection with the exception of Ibafect and siRNA transfection, where only a similar trend was observed (Fig 1A–1D). The significant proliferation decreases varied between 39.9% and 85.8% compared to controls, with combined PEI/FluidMag being the most effective (p = 2.5x10^-4^, N = 4, Fig 1D). Notably, transfection of naPBMCs not containing the phagocytotic monocyte subpopulation, showed more pronounced proliferation inhibition compared to PBMCs (Fig 1A/1B vs. 1C/1D for AON or siRNA transfection).

Cell viability was measured by Trypan blue staining, a dye which only penetrates into necrotic cells due to their impaired membrane barrier. Polycationic PEI continuously reduced viability significantly in comparison to untreated control cells (p_PBMC_ < 2 x 10^−5^, p_na-PBMC_ < 3 x 10^−4^, N = 4, Fig 1E). In contrast, transfection with or without magnetic enhancement did not differ in terms of viability between treated samples. Regardless of the applied transfection reagent or method, oligonucleotide uptake rates did not differ significantly and remained at about 30–50% (Fig 1F). Interestingly, amounts of fluorescence labeled nucleic acids transfected into cells did indeed differ significantly dependent on iron oxide particle treatment. In cells additionally treated with iron oxide nanoparticles for enhanced transfection, an increase of average amounts of oligonucleotide taken up was observed as compared to cells transfected without enhancement. This was significant for Ibafect in PBMCs and na-PBMCs, and for PEI in na-PBMCs, whereas in PBMCs only a trend was detected (S1 Fig).

### Cell cycle is affected by transfection methods

**Fig 2:**
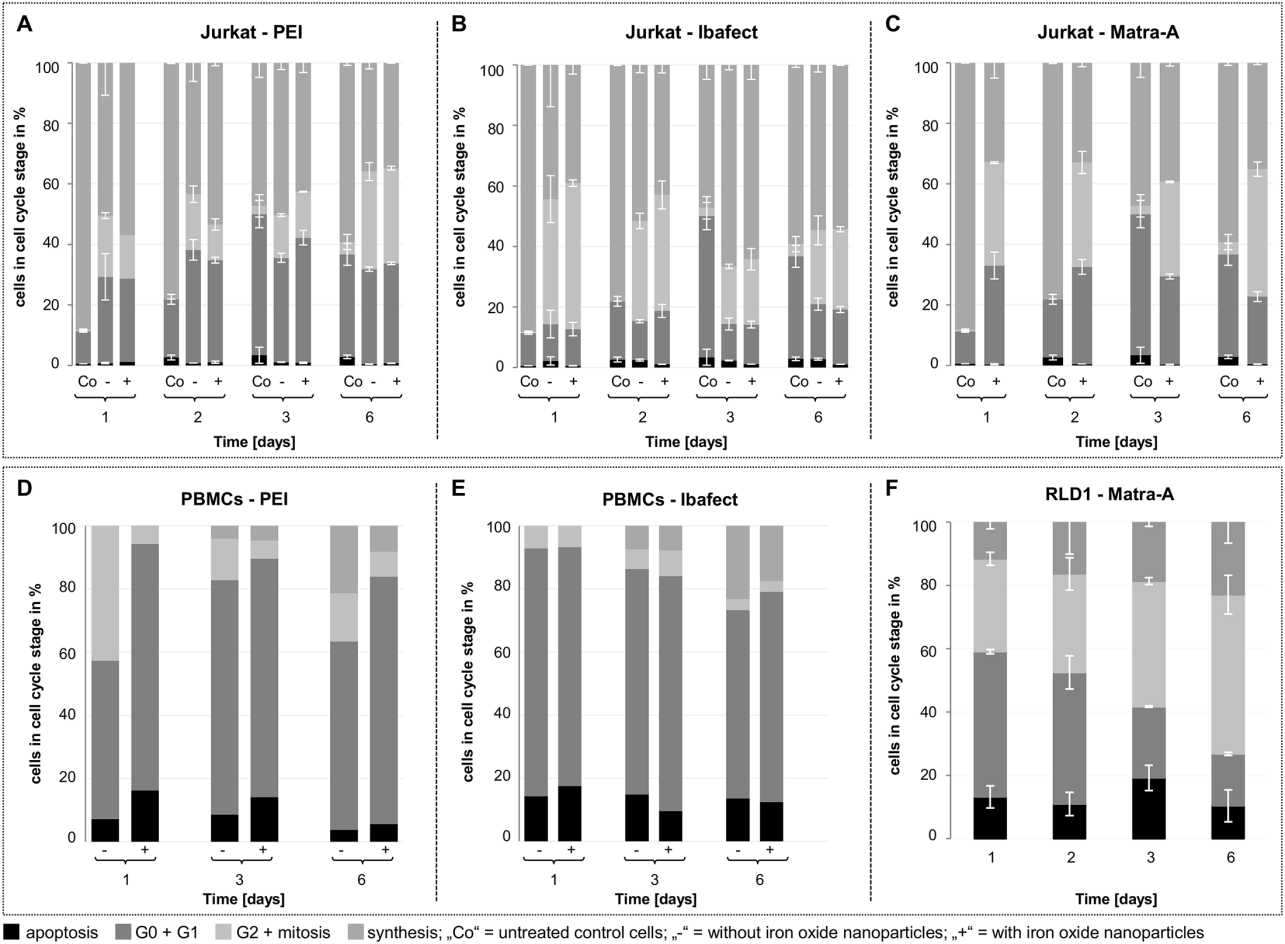
Cell cycle analysis of nanoparticle-transfected lymphocytes. Cells were transfected with nonsense FAM-AON using cationic polymer particles (A/D: PEI) +/- magnetic beads (FluidMag) or liposomal particles (B/E: Ibafect)) +/- magnetic beads (MA Enhancer) or iron oxide particles alone (C/F: Matra-A) for 24h, followed by persistent PHA stimulation (0.05 mg/ml) for up to 6 days. Human Jurkat T cells were transfected as duplicates (A-C), human PBMCs prepared from one donor (D/F) and murine RLD1 T cells were transfected as duplicates as well (F). Shown is the mean percentage of cells in a given cell cycle stage with error bars indicating SD. The APC BrdU Flow kit (BD bioscience) was used to assess the percentage of cells in a distinct cell cycle phase by flow cytometry on days 1, 2, 3 and 6 after transfection. For the T cell lines Jurkat and RLD1, not transfected samples (“controls” = Co) were also monitored for BrdU incorporation on days 1, 2, 3 and 6.

Next, we assessed whether the inhibitory effect on proliferation of immune cells seen in the ^3^H-thymidine genomic integration assay correlates with changes in cell cycle stages. Here, the magnetic bead-mediated enhancement of antisense oligonucleotide transfection with polycationic PEI increased the percentage of resting G0/G1 cells, while mitosis was reduced within 24h post transfection in Jurkat cells as well as PBMCs (Fig 2A/2D). This effect was present up to day 2 in Jurkat cells and day 6 for PBMCs. On day 3, a significant increase in resting cells was observed in samples transfected with PEI and iron oxide particles compared to samples transfected with PEI alone. In contrast, when using Ibafect cell cycle stages did not differ much between PBMCs and Jurkat cells treated with or without magnetic enhancement (Fig 2B and 2E). While in the case of PEI enhanced by magnetic beads this correlates well with our findings of decreased proliferation detected by LTT, the absence of effects on cell cycle in the case of Ibafect was in contrast to its anti-proliferative effect.

To assess the effect of magnetic nanoparticles alone, we also transfected Matra-A beads. Here, we used two proliferating tumor T cell lines Jurkat (human) and RLD1 (murine) because primary cells showed insufficient uptake rates with this method (data not shown). Additionally, results for Matra-A treatment of Jurkat support data shown for Matra-A in RLD1 T cells. In Jurkat cells, we observed an initial, similar increase in the percentage of resting cells (G0/G1) that later on decreased again until reaching a minimum on day 6, while in parallel the percentage of cells in mitosis increased to maximum levels (Fig 2C and 2F). Upon addition of magnetic beads, we detected a significant decrease of cells in S phase and an increase of cells in G0/G1 at 24h post transfection (Fig 2C/2F). Within the following days, the percentage of cells in mitosis increased significantly. Concomitantly, the percentage of resting cells (G0/G1) decreased again up to day 6. For all samples, share of apoptotic cells was comparable to literature (below 5% for Jurkat, below 20% for primary PBMCs and RLD1) and differences between treatments were minimal.

### mRNA expression of proliferation genes is affected by nanoparticle-mediated transfection

**Fig 3:**
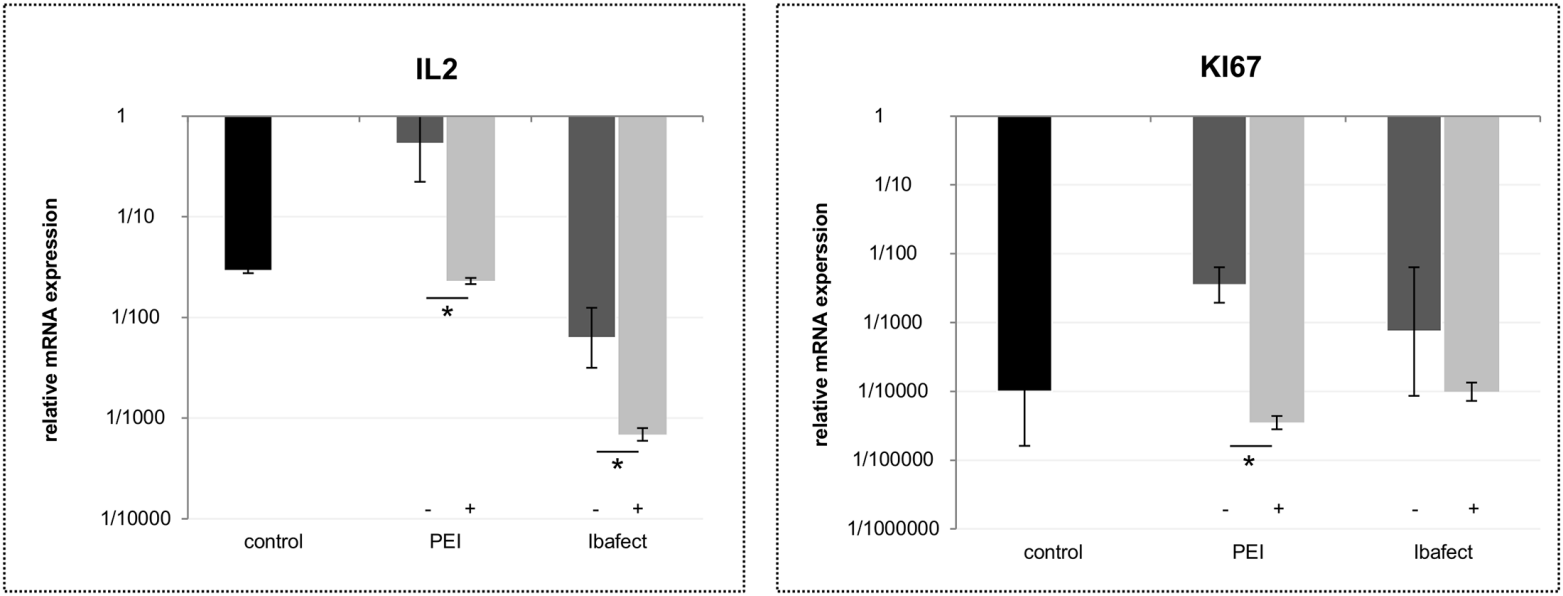
Proliferation-gene expression in PBMCs transfected with AON w/o iron oxide nanoparticles (-); with particles (+). PBMCs (N_donor_ = 4) were transfected with AON with or without magnetic iron oxide nanoparticle transfection enhancement by applying PEI (+/- FluidMag) and Ibafect (+/- MA Enhancer) for 24h, followed by persistent PHA stimulation (0.05 mg / ml) for 72h. RT-qPCR in quadruplicates per donor was performed for measuring *Ki67* and *Il2* mRNA expression. Data were normalized to housekeeping Rplp0 mRNA expression and expression is shown relative to housekeeping expression. Fold changes were calculated using the ΔΔCt method and are represented as mean ± SE. *p<0.05 by Student´s T Test.

The mRNA expression of genes involved in proliferation was influenced by nanoparticular transfection as well. Interestingly, PEI transfection without magnetic enhancement increased the expression of *Il2* (p = 0.003, N = 4), while LTT results did not show proliferation stimulating effects (Figs 3 vs. 1A). In contrast, increased proliferation rates by Ibafect as measured by LTT were not accompanied by significant changes in *Ki67* and *Il2* expression compared to untreated control cells (Figs 3 vs. 1A).

But reduced *Il2* expression upon addition of magnetic beads correlated well with LTT results for PEI as well as Ibafect (p = 0.002 and p = 0.007, respectively, N = 4). The expression of *Ki67* was also significantly reduced in samples treated with PEI and beads compared to only PEI treated samples (p = 0.002, N = 4). A similar trend was observed for Ibafect but did not reach significance (Figs 3 vs. 1A). Thus, the expression of *Il2* and *Ki67* might generally be inverse related to proliferation within the observed experimental time frame.

### Prolonged survival in a murine model of GvHD upon *ex vivo* transfection with nanoparticles containing a non-specific AON

**Fig 4:**
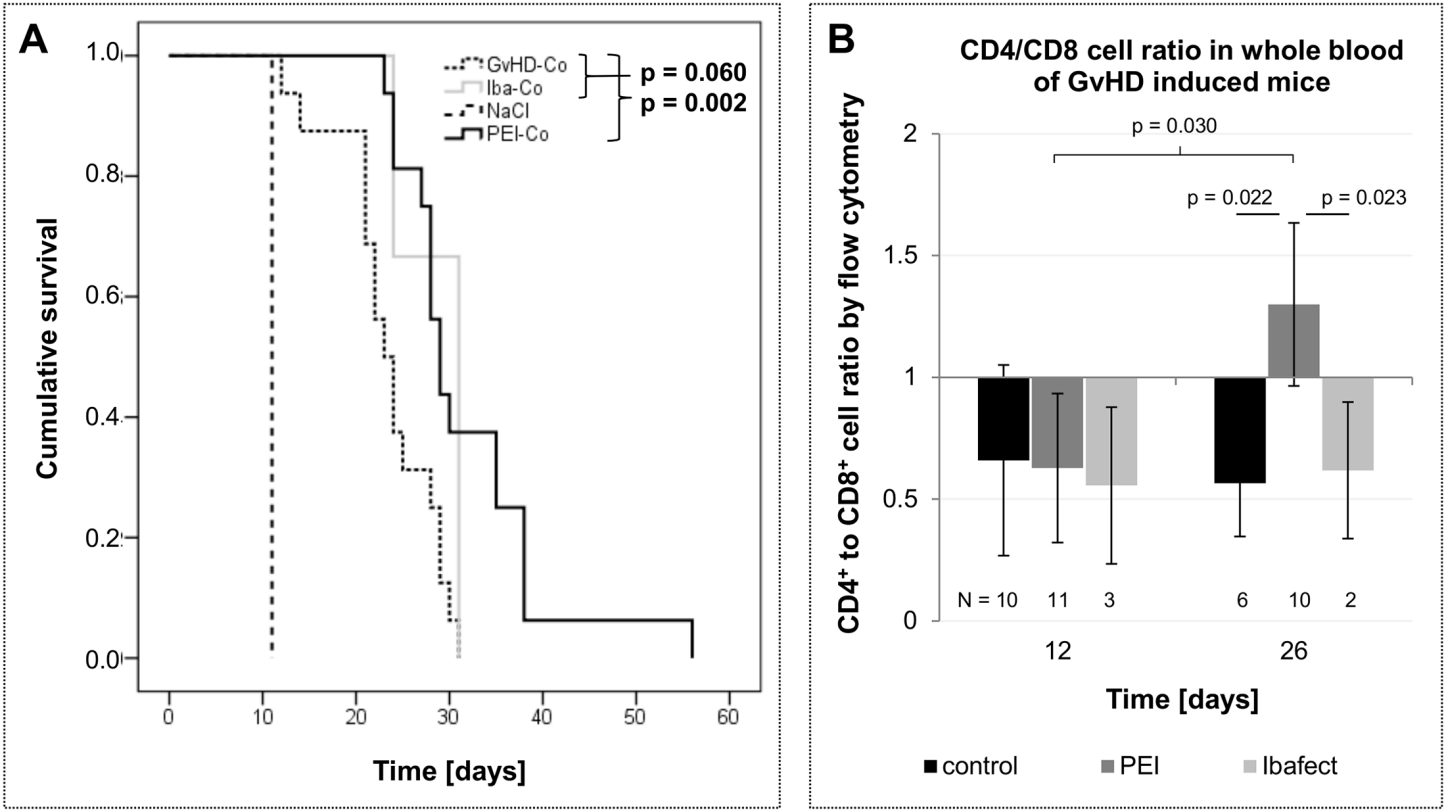
Ex vivo transfection of nanoparticles in a murine model of GvHD. Irradiated Balb/c^wt^ mice (0.4 Gy/g, max. ≤ 8 Gy) were transplanted with splenocytes and bone marrow cells (2 x 10^7^ cells each in 150 μl 0.9% NaCl, applied i.v. via tail vein) from donor C57bl/6^wt^ mice. The transplants were either untreated (GvHD-Co, N = 16) or transfected with 10 μg nonsense antisense oligonucleotide (2’O-methyl-PTO-RNA) and PEI (PEI-Co, N = 16) or Ibafect and MA Enhancer (Iba-Co, N = 3) for 4h. Irradiation control mice were not transplanted with cells, but injected with 0.9% NaCl (NaCl, N = 4). (A) Cumulative survival is shown as a Kaplan Meier curve (calculated by SPSS, vs. 22) and significance was calculated by Log-Rank (Mantel-Cox)-Test. Shown p-values refer to survival of transfected animal group compared to control group (GvHD-Co). (B) Shown are mean ratios of CD3^+^/CD4^+^ to CD3^+^/CD8^+^ double positive cells as detected by flow cytometry in retro-orbital blood collected at days 12 and 26 and error bars indicate SD. Blood was treated with BD FACS Lysing Solution prior to staining with anti-CD3e-FITC (Cat. No. 553062), anti-CD4-PE-Cy7 (Cat. No. 552775) and anti-CD8-PerCP (Cat. No. 553036) flow cytometry antibodies. Significance was calculated by Student’s T test.

In the *in vivo* GvHD disease model, murine splenocytes containing primary immune cells were transfected with a non-specific AON using PEI (PEI-Co, N = 16) or Ibafect/MA Enhancer (Iba-Co, N = 3) prior to transplantation into irradiated host animals. A marked increase in survival was observed upon transplantation vs. untreated mice (Fig 4A). Notably, compared to hosts receiving untreated transplants (GvHD-Co, N = 16), the treated groups showed a further increase in survival which in the case of PEI reached statistical significance (p_PEI_ = 0.002; p_Ibafect_ = 0.060) (Fig 4A). This is in line with our results from the LTT assay demonstrating significantly reduced proliferation of splenocytes upon transfection with PEI (1036 +/- 322 ccpm vs. 13332 +/- 2439 ccpm, p = 0.012), and thus corroborates the notion that PEI is able to induce prolonged survival possibly due to its anti-proliferative effects.

We also studied the ratio of CD4^+^ to CD8^+^ cells, an indicator of immune activity in the course of GvHD. In healthy untreated host animals, this ratio was 3.6 +/- 0.4 (N = 10), which correlates to findings in healthy humans. Following irradiation, stem cell transplantation and the onset of GvHD, this ratio was reversed. On day 12, when hematopoiesis just began to recover, all groups showed a ratio in favor of CD8^+^ cells without significant differences detectable between groups (Fig 4B). In contrast, following the onset of GvHD after 26 days, significant differences in favor of CD4^+^ cells were detectable in PEI treated animals (p_vs.control_ = 0.022, p_vs.Ibafect_ = 0.023, p_vs. PEI, day 12_ = 0.03; N_control, day 26_ = 6, N_Ibafect, day 26_ = 2, N_PEI, day 12_ = 11, N_PEI, day 26_ = 10), but not in untreated controls or upon Ibafect treatment.

## Discussion

For the first time, we present data on the effects of nanoparticle-based transfection of immune cells on their mitogenic proliferation. In case of immune cells, proliferation of effector cells is a fundamental process within the adaptive immune system, since expansion of immune cell populations is important for the maintenance of cell numbers in the periphery (homeostatic proliferation) to properly represent naïve and memory cells for continued diversity [26]. Furthermore, proliferation upon antigen contact is an essential step in the immunological response to infection. Consequently, reagents or processes that reduce immune cell proliferation can cause destabilization of the immune cell composition important for homeostasis. On the other hand, proliferation also needs to be highly regulated to prevent inappropriate activation of immune cells resulting in chronic inflammation [27], autoimmune or allergic disorders [28].

In this study, we show that the transfection with polymer- or lipid-based nanoparticles influence proliferation of immune cells. We applied Lipofectamine^®^ 2000 and Ibafect for liposomal, and polyethylenimine (PEI) for polycationic transfection according to the nomenclature of synthetic gene delivery systems [29]. These transfection reagents are widely utilized in molecular biology for transient or stable transfection showing high transfection efficiency and low cytotoxicity, and some of them have also been explored *in vivo* in animal models and in clinical trials [30–36]. Additionally, magnetic iron oxide nanoparticles are a promising approach for efficient non-viral *in vitro* and *in vivo* gene therapy. In the scientific literature, ‘magnetofection’ is a generic term for magnetically guided and enhanced nucleic acid delivery [37]. Beyond this, magnetic nanoparticles offer an even wider range of applications, for example, as a systemic drug delivery system or for the tracking of labeled cell therapy products by magnetic resonance imaging (MRI) [38–40]. For example, labeled stem cells have become a valuable tool in the understanding and evaluation of experimental stem cell-based therapies [41]. However, the systemic administration of magnetic nanoparticles could non-specifically influence basic cellular functions and engage defense mechanisms against foreign particles or molecules [37]. Nanoparticles might also be tagged by the complement system and eliminated by phagocytotic immune cells [42–44].

Previous studies investigated the biodistribution, toxicity and transfection efficacy of nanoparticles and often showed them to be non-toxic at effective dosage levels [45]. However, the impact of nanoparticles on the immune system and the specific functions of immune cells were not considered in these studies.

While we could confirm that the transfection methods applied in our study did mostly not induce apoptosis at standard dosages, we found substantial effects on cellular proliferation. In particular, we demonstrate a general proliferation inhibiting effect upon utilization of magnetic iron nanoparticles for transfection enhancement. This effect was also most likely independent off any influence on general viability, as there was no significant difference between iron oxide nanoparticle transfection enhanced samples compared to samples transfected but not enhanced. In contrast, liposomal or polycationic nanoparticles without magnetic bead assistance showed divergent effects, with liposomal Lipofectamine and Ibafect stimulating proliferation of AON transfected PBMCs in contrast to polycationic PEI. Interestingly, there might be a correlation between proliferation inhibition and average amounts of oligonucleotide taken up per cell, with cytosolic overload potentially playing a role. Notably, PEI also markedly inhibited proliferation of murine splenocytes, a finding relevant for pre-clinical animal studies.

Analysis of cell cycle stages following transfection indicated cell cycle arrest as one underlying mechanism associated with the anti-proliferative effect mediated by addition of magnetic particles. These results were further corroborated by a significant decrease in *Il2* expression, a cytokine closely associated with T cell proliferation [46], upon addition of magnetic beads.

To analyze whether the anti-proliferative effect mediated by nanoparticles can be transferred to a clinically more relevant *in vivo* setting, we applied *ex vivo* transfected stem cells in a murine model of graft versus host disease (GvHD). In GvHD, immune cells within donor tissues confer pathological systemic immune activation directed against host tissue. It is known that modulation of donor T cells or T cell subsets, e.g. by T cell specific antibodies, prevents development of GvHD [22]. It is also known, that GvHD is mainly conferred by T cells and a hallmark of T cell activation is increased T cell proliferation and expression of proliferation signals such as Il2. Additionally, van Leeuwen et al. specifically reported on our GvHD model that donor cell proliferation and Il2 expression occurred within the first 2–3 weeks [47]. Thus, any significant delay of GvHD might be reasonably attributed to decreased donor T cell activation and proliferation.

Here, PEI nanoparticles that previously showed anti-proliferative effects *in vitro* significantly prolonged survival and reduced pro-inflammatory predictors (p = 0.002). Concomitantly, the combined magnetofection method Ibafect/Enhancer prolonged survival (p = 0.06) in our animal model as well. The fact that in the latter case no statistical significance was reached could be due to relatively small animal numbers within the experiment, or could be associated with generally somewhat smaller anti-proliferative effects on murine immune cells by the liposomal Ibafect in contrast to polycationic PEI. In the future, it would also be prudent to apply cell tracking methods to investigate *in vivo* cell proliferation directly.

While no prior study investigated nanoparticle-mediated inhibition of proliferation in immune cells, McMahon et al. previously showed a reduced formation of LPS stimulated B lymphoblasts upon transfection with the polymer DEAE-dextran [15]. Although they did not further investigate the underlying cause of this effect, it is reasonable to assume that an unspecific anti-proliferative effect of the transfection reagent might have been present.

So far, a possible causative link between magnetofection, inhibited proliferation and arrested cell cycle is still unclear. While complexes of PEI/nucleic acid and PEI-FluiMag/nucleic acids showed no significant differences during endocytosis [48], magnetic particles applied to immobilized target cells induce a strong nanoparticle-cell contact during sedimentation in a magnetic field. While this improves transfection efficiency, it could also cause a temporary inhibition of cell proliferation as endocytosis and the formation of the spindle apparatus at the beginning of mitosis are connected processes [49]. It is known that cells reduce endocytotic activity at the beginning of mitosis [50] and it was demonstrated that membrane tension of cells entering mitosis rises while endocytosis rate decreases and vice versa [51]. Furthermore, by applying amphiphilic compounds the endocytosis rate was enhanced due to reduced membrane tension [51]. Thus, increased endocytotic activity after applying iron oxide magnetic nanoparticles to cells in a magnetic field could lead to a decrease in membrane tension, preventing cell entry into mitosis and therefore reducing proliferation rates as seen in our study.

Taken together, our results indicate an unspecific pro- or anti-proliferative, and therefore pro- or anti-inflammatory effect could be conferred by nanoparticle-mediated transfection. It might be possible to utilize this effect to enhance a desired therapeutic approach in one way or the other. *In vivo* transfection approaches, however, will also require analysis to what extent anti-proliferative effects would also affect other, non-immune system related cell types. In case of gene therapy targeting highly proliferative cancer cells, an auxiliary anti-proliferative effect mediated by nanoparticles further increase the therapeutic outcome.

## Supporting information

S1 Fig Median amount of intracellular FL-labeled siRNA. PBMCs or non-adherent PBMCs (naPBMCs) (N = 4) were transfected with Alexa488 labeled nonsense siRNA with or without magnetic iron oxide nanoparticle transfection enhancement (dark gray = without (-), light gray = with particles (+)) by applying PEI (+/- FluidMag) and Ibafect (+/- MA Enhancer). Controls were untransfected cells (black bar). Uptake of labeled siRNA was measured by flow cytometry as a function of fluorescence intensity. Median FL intensity ± SE is shown.
(TIF)
