# Supplementary figures and images for: Influence of nanoparticle-mediated transfection on proliferation of primary immune cells in vitro and in vivo
Source: PLoS One. 2017 May 2;12(5):e0176517. doi: 10.1371/journal.pone.0176517 (PMC5412997; doi:10.1371/journal.pone.0176517)

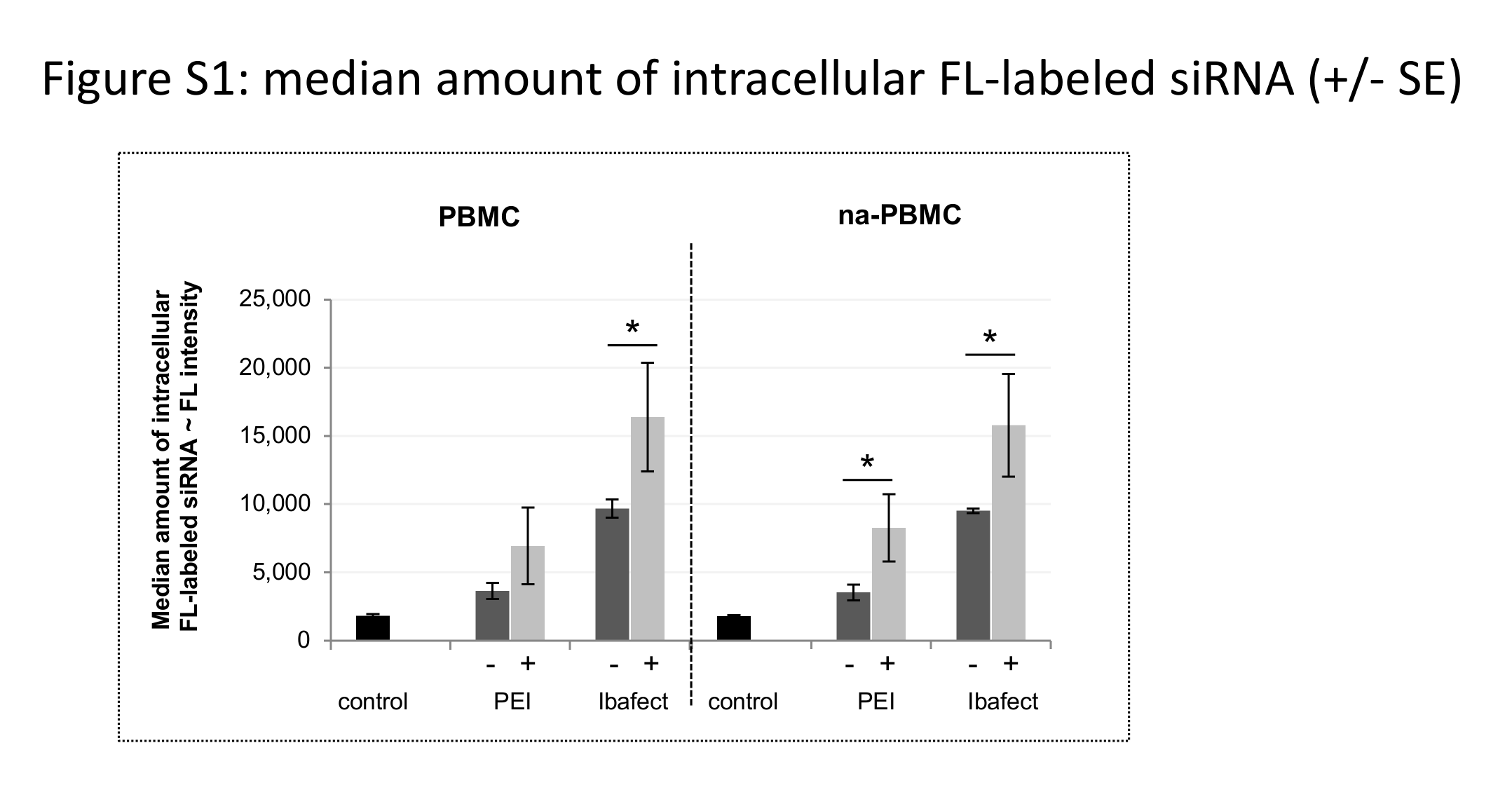

Supplement: S1 Fig — PBMCs or non-adherent PBMCs (naPBMCs) (N = 4) were transfected with Alexa488 labeled nonsense siRNA with or without magnetic iron oxide nanoparticle transfection enhancement (dark gray = without (-), light gray = with particles (+)) by applying PEI (+/- FluidMag) and Ibafect (+/- MA Enhancer). Controls were untransfected cells (black bar). Uptake of labeled siRNA was measured by flow cytometry as a function of fluorescence intensity. Median FL intensity ± SE is shown. (TIF) [file pone.0176517.s001.tif]
